# Drug resistance and genomic variations among *Mycobacterium tuberculosis* isolates from The Nile Delta, Egypt

**DOI:** 10.1038/s41598-024-70199-8

**Published:** 2024-09-02

**Authors:** May S. Soliman, Chungyi H. Hansen, Mostafa Hanafy, Sherine Shawky, Heba Rashed, Mohamed Abdullah, Noha Salah Soliman, Maha A. Gad, Sahar Khairat, Amani El-Kholy, Adel M. Talaat

**Affiliations:** 1 Department of Clinical and Chemical Pathology, Kasr Al Aini, Faculty of Medicine, Cairo University, Giza, Egypt; 2 Department of Pathobiological Sciences, University of Wisconsin-Madison, Madison, USA; 3 Microbiology Department, Medical Research Institute, Alexandria University, Alexandria, Egypt; 4 Clinical Pathology Department, Faculty of Medicine, Assiut University, Assiut, Egypt; 5 Central Public Health Laboratories, Ministry of Health and Population, Cairo, Egypt; 6 Department of Microbiology and Immunology, Faculty of Veterinary Medicine, Cairo University, Giza, Egypt

**Keywords:** Tuberculosis, Genome-wide variations, Drug resistance, Lineages, Egypt, Tuberculosis, Infectious-disease diagnostics

## Abstract

Tuberculosis is a global public health concern. Earlier reports suggested the emergence of high rates of drug resistant tuberculosis in Egypt. This study included 102 isolates of *Mycobacterium tuberculosis* collected from two reference laboratories in Cairo and Alexandria. All clinical isolates were sub-cultured on Löwenstein–Jensen medium and analyzed using both BD BACTEC MGIT 960 SIRE Kit and standard diffusion disk assays to identify the antibiotic sensitivity profile. Extracted genomic DNA was subjected to whole genome sequencing (WGS) using Illumina platform. Isolates that belong to lineage 4 represented > 80%, while lineage 3 represented only 11% of the isolates. The percentage of drug resistance for the streptomycin, isoniazid, rifampicin and ethambutol were 31.0, 17.2, 19.5 and 20.7, respectively. Nearly 47.1% of the isolates were sensitive to the four anti-tuberculous drugs, while only one isolate was resistant to all four drugs. In addition, several new and known mutations were identified by WGS. High rates of drug resistance and new mutations were identified in our isolates. Tuberculosis control measures should focus on the spread of mono (S, I, R, E)- and double (S, E)-drug resistant strains present at higher rates throughout the whole Nile Delta, Egypt.

## Introduction

Infection with *Mycobacterium tuberculosis* (*M. tuberculosis*) affects one quarter of the world population, causing nearly 1.3 million deaths/year according to WHO estimates from 2022^1^. Additionally, the emergence of drug resistant (DR) and multi-drug resistant (MDR) isolates of *M. tuberculosis* is on the rise globally^2^, including in Egypt^3^. In the Middle East, drug-resistant isolates of *M. tuberculosis* are understudied^4,5^. Analysis of a large number of *M. tuberculosis* isolates collected from different countries around the globe identified resistance to isoniazid (katG mutations) as a precursor for the emergence of MDR-TB and other more complex drug resistance profiles^6^. Unfortunately, there were no isolates in this study from the Middle East, despite the inclusion of 8316 isolates from other areas. In Egypt, tuberculosis is considered a significant national health problem by public health authorities^7^. Earlier studies using growth-based phenotyping methods^5,8^ or a combination of geno- and genome-typic approaches^9,10^, all indicated the emergence of primary drug resistance among patients in the Middle East. A recent study from our group revealed high rates of drug resistance in Egypt reaching up to 35%^11^.

An important consequence of the COVID-19 pandemic has been a worsening of the TB epidemic globally with the first time increase in reported tuberculosis infection to ~ 7.5 million since the start of WHO global monitoring^1^. The burden of drug-resistant tuberculosis (DR-TB) also increased by 3% between 2020 and 2021, with 450,000 new cases of rifampicin-resistant TB (RR-TB) in 2021 alone. In Egypt, tuberculosis incidence rate fell from 15,000 cases per 100,000 people in 2015 to only 11 cases in 2021^12^. Whether this decrease is real or represents under-reporting remains unclear, although it follows global trends resulting from the impacts of the COVID-19 pandemic. In a retrospective study on TB patients in Egypt from 2014 to 2018, the majority were young, middle-aged male; and the rates of relapse and therapeutic failure were 3.1% and 3.9%, respectively^13^.To our knowledge, the effect of the COVID-19 pandemic on TB drug resistance has not been reported from Egypt, though Egypt experienced a significant rise in antimicrobial resistance (AMR) in other bacteria during the pandemic between 2020–2021^14^, the same period targeted for analysis in this report.

The main objectives of this report include profiling the genome-wide lineages and drug resistance phenotypes/genotypes of *M. tuberculosis* isolates circulating among patients in Egypt. Interestingly, almost 5% of the clinical isolates from southern region of the Nile Delta were *M. bovis* with potential zoonotic importance while another ~ 9% belonged to several members of non-tuberculous mycobacteria (NTM). The presence of both *M. bovis* and NTM isolates among human samples highlights the importance of zoonotic and environmental mycobacteria in human infections. The majority (> 80%) of *M. tuberculosis* isolates belonged to lineage 4 of *M. tuberculosis*. More importantly, mono-drug resistant isolates ranged from 17.2 to 31.0% while the double drug resistant reached 10% of the total isolates in addition to 4.5% of isolate have meet the criteria for multi-drug resistant (MDR) set forth by the WHO^15^. Finally, genome-wide sequencing identified 100% of the known single nucleotide polymorphism (SNPs) associated with ethambutol and other antibiotics with varying degrees, providing new candidate SNPs for the four front-line antibiotics used to treat tuberculosis (S, I, R, E).

## Materials and methods

### Ethical statement

The study was approved both by the Research Ethics Committee of the Faculty of Medicine, Cairo University and the Ministry of Health and Population, Egypt, and by the University of Wisconsin-Madison’s Institutional Review Board. All methods were performed in accordance with the Office of Biological Safety guidelines and regulations. Informed consent was waived by Institutional Review Board of the University of Wisconsin-Madison as we used archived, de-identified mycobacterial isolates. All isolates were de-identified to maintain patient privacy. The study was conducted jointly in the Mycobacteriology Laboratory and the Infectious Diseases Molecular Research Laboratory of Cairo University Hospital in Egypt as well as the Laboratory of Infectious Genomics, at the University of Wisconsin-Madison in the USA.

### Isolates collection and culturing

During the study period (March 2020 and February 2021), Cairo central laboratory at Kasr Elaini Hospital collected and analyzed 102 isolates initially identified as *M. tuberculosis* from different geographically areas within the Nile Delta, Egypt. Cairo central laboratory received isolates from hospitals serving Cairo, Giza and Kaliobia governorates (referred to here as South Delta region) while Alexandria central laboratory received isolates from Alexandria, Beheira, Rasheed, Kafr El-Sheikh governorates (referred to here as North Delta region). All clinical isolates were initially examined by acid-fast staining and were from both pulmonary and extra pulmonary specimens. Isolates were sub-cultured on Löwenstein–Jensen (LJ) medium slants (DB Difco, New York) to obtain fresh colonies for further antibiotic sensitivity testing and for genomic DNA extraction. All isolates were shared and processed at both Cairo and Wisconsin Universities.

### Antibiotic sensitivity testing

For phenotypic analysis, antibiotic sensitivity testing (AST) of the isolates was conducted using automated proportion method using the BD BACTEC MGIT 960 SIRE Kit (BD, Franklin Lakes, NJ) operated on the MGIT 960 instrument system (BD) following the manufacturer’s instructions (BD) as detailed before^11^. For the standard plate assay, anti-tuberculosis drugs were added to Middlebrook 7H10 agar plates, pouring 5 ml per quarter of a four quadrant plate. The final concentration of each antibiotic was ~ 10 µg of streptomycin, 0.2 µg of isoniazid (INH), 5 µg of rifampicin (RMP), and 5 µg of ethambutol (EMB), or left free of antibiotic as a positive control. Only three anti-tuberculosis drugs were determined in each plate to allow for the antibiotic free control. *M. tuberculosis* H37Rv is also included as a drug susceptible control with each batch of AST. Plates were inoculated with the 25 µl of each isolate per quadrant, directly or serially diluted from early log phase cultures grown in Middlebrook 7H9 broth (BD) to an OD_600_ of 0.2–0.3 and incubated at 37 °C for 1–3 weeks^11^. Results reported here is the combination of both BACTEC system and the standard plate assay.

### Genomic DNA extraction and isolation

Genomic DNA was extracted from all *M. tuberculosis* isolates using QIAamp^®^ DNA mini kit from Qiagen^16^ and as outlined before by our group^11,17^. Briefly, a loopful of mycobacterial culture was resuspended in equal volume of TE buffer. Bacterial suspensions were placed at 80 °C for 20 min to kill all living organisms. Tubes were allowed to cool at room temperature and 10 µl of 100 mg/ml lysozyme was added to each tube followed by incubation at 37 °C for 3 h with occasional mixing. All subsequent steps for DNA extraction and purification were conducted according to the manufacturer protocol. Quality of the gDNA was verified by both NanoDrop (Thermo Scientific, Wilmington, DE) machine and fluorometer (Denovix).

### Multiplex-PCR phylogenetic analysis

A multiplex PCR targeting major Mycobacterium species was performed as outlined before (https://pubmed.ncbi.nlm.nih.gov/28659320/)^18^. This PCR differentiates *M. tuberculosis* complex (MTBC) and non-tuberculosis mycobacteria (NTM). It also further distinguishes *M. tuberculosis* from other members of the *M. tuberculosis complex* (MTBC), as well as distinguish five clinically relevant NTM (*M. avium*, *M. intracellulare*, *M. abscessus*, *M. massiliense*, and *M. kansasii*) using mtbk_20680 sequence as a conserved target among isolates, including the Beijing lineage. Eight sets of primers were included in one reaction. *M. tuberculosis* H37Rv, *M. tuberculosis* Beijing strain and *M. avium subsp. avium* were added as controls.

### Whole genome sequencing

The extracted genomic DNA was used for whole genome bacterial sequencing using Illumina MiSeq platform at Kasr Al Ainy Sequencing Lab and the University of Wisconsin-Madison Biotechnology Center. A total of bacterial DNA (1 ng) was used in the library preparation using Nextera XT DNA Library preparation kit (FC-131-1096, Illumina, San Diego, CA, USA), according to the manufacturer’s instruction. Briefly, transposons were used to fragment the DNA, subsequently adapter sequences were added onto the DNA template, products were size-selected for optimum insert length, enriched and quantified. Sequencing was carried out with the MiSeq reagent kit 600 v3 (Illumina, San Diego, CA, USA) on the Illumina MiSeq, generating an average of 301 base pair paired-end reads^17,19^.

CLCBio Genomic Workbench was used to run reference assembly against *M. tuberculosis* H37Rv (NC_000962.3) with variant calling. Consensus sequences were extracted from all samples to build a phylogenetic tree using Harvest suite and ParSNP^20^ was used to align all sequences with an approximately-maximum-likelihood method taking into consideration all SNPs, indels, and structural differences found in the genomes. For other bioinformatic analysis, fastQ files were submitted to Mykrobe^21^ and TB profiler online tools^22^ to determine the lineage, family, spoligotype and to determine the mutations conferring resistance to anti-tuberculosis drugs. All sequences had at least 50× coverage designation of the mutation as well as visualization of the alignment.

## Results

### Genotypic analysis of *M. tuberculosis* from the Nile Delta

This study included 102 mycobacterial isolates collected from Greater Cairo and the northern and southern regions of the Nile Delta, Egypt (Supplementary Fig. 1), among which, only 93 isolates were able to grow reproducibly on LJ slants. The majority (88%) of the isolates were obtained from pulmonary sample sources including broncho–alveolar lavage, pleural fluid and sputum samples, while only 12% were obtained from extra-pulmonary samples including pus, tissue and urine. To confirm strain identity and genotype, multiplex PCR was able to confirm that majority of isolates (87, 93.5%) belong to *M. tuberculosis* complex (MTBC) while NTM represented 5.3% of the isolates (Supplementary Fig. 2). Interestingly, isolates numbers 36, 52, 56, and 74 were found to belong to Beijing family based on the multiplex PCR results^18^. Among the MTBC members, isolates belong to the *M. bovis/BCG* were identified in ~ 5% of the isolates. Identities for members of the MTBC and NTM were further analyzed by WGS analysis.

### Whole genome sequence analysis

**Figure 1:**
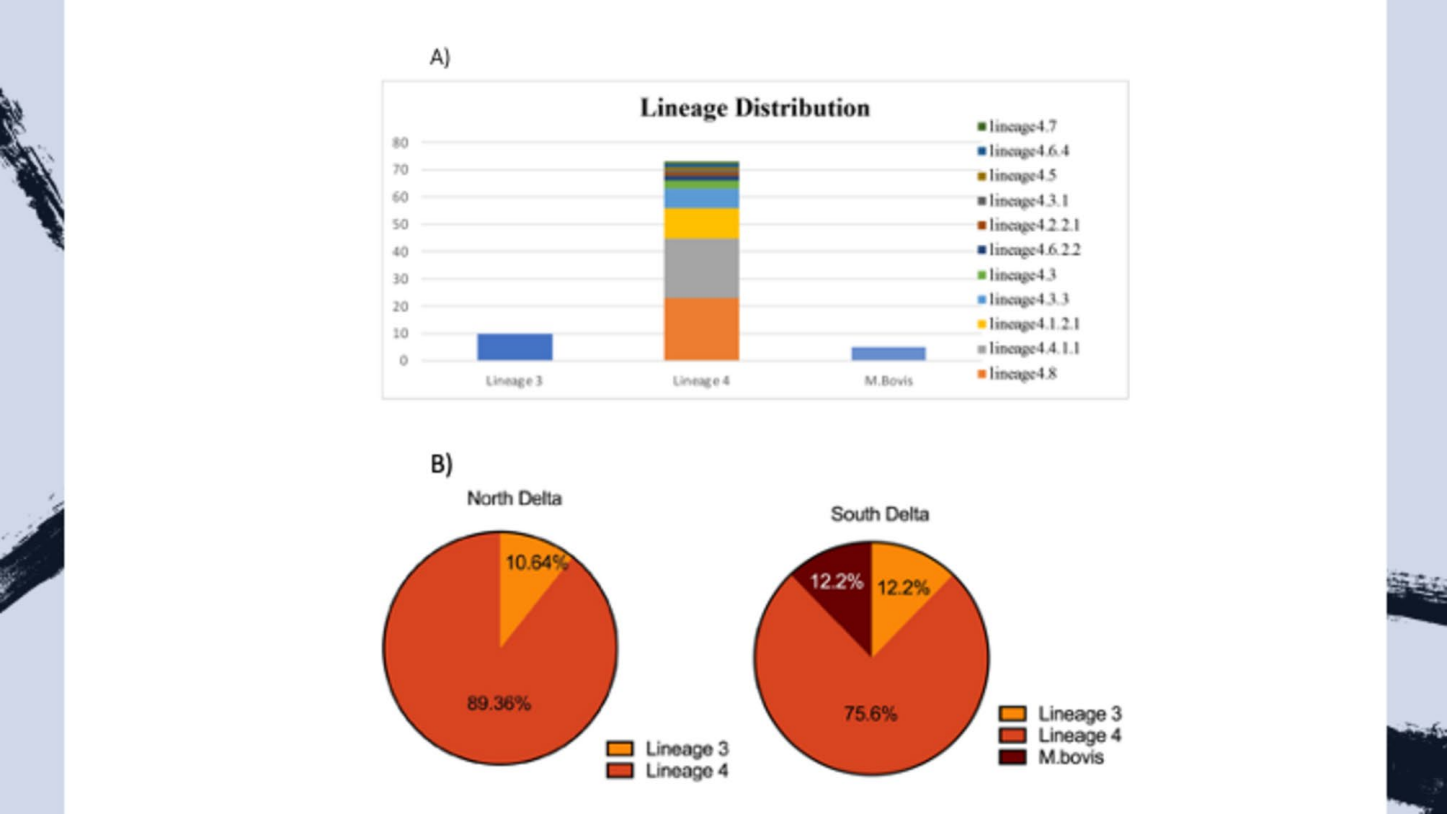
*M. tuberculosis* lineages identified in the in both North and South Nile Delta region during 2020–2021, Egypt. (**A**) Lineage and sub-lineage distribution among *M. tuberculosis* isolates. (**B**) Geographical distribution of mycobacterial lineages among North (Alexandria central laboratory) and South (Cairo central laboratory) Delta regions.

To further decipher isolate genotype**,** Illumina MiSeq next generation sequencing was performed on all grown 87 isolates belonging to *M. tuberculosis* complex (MTBC). Using bioinformatics tools of Mykrobe and TB Profiler, we identified 83.9% sequenced genomes belong to lineage 4 while 11.4% belong to lineage 3 (Fig. 1A). Moreover, five isolates were identified as *M. bovis,* a strain that is mainly isolated from animals but can infect humans (a zoonotic mycobacteria)^23^. For lineage 4 (Euro–American Family), sub-lineage 4.8 was most predominant (26.2%), followed by 4.4 (25%), then 4.1 and 4.3 (12.5% each). All lineage 3 corresponded to the subtypes of (East-African–Indian family), the Central Asian spoligotypes (CAS), and RD 750 (Supplementary Table 1). Interestingly, 12% of isolates belonged to the zoonotic *M. bovis* isolate located only in the South Delta (Fig. 1B). However, isolates 52 and 56 showed as lineage 3 using Mykrobe and TB profiler platform while Beijing strains belong to lineage 2 based on multiplex PCR.

### Phylogenetic analysis of sequenced samples comparison with known *M. tuberculosis* strains

**Figure 2:**
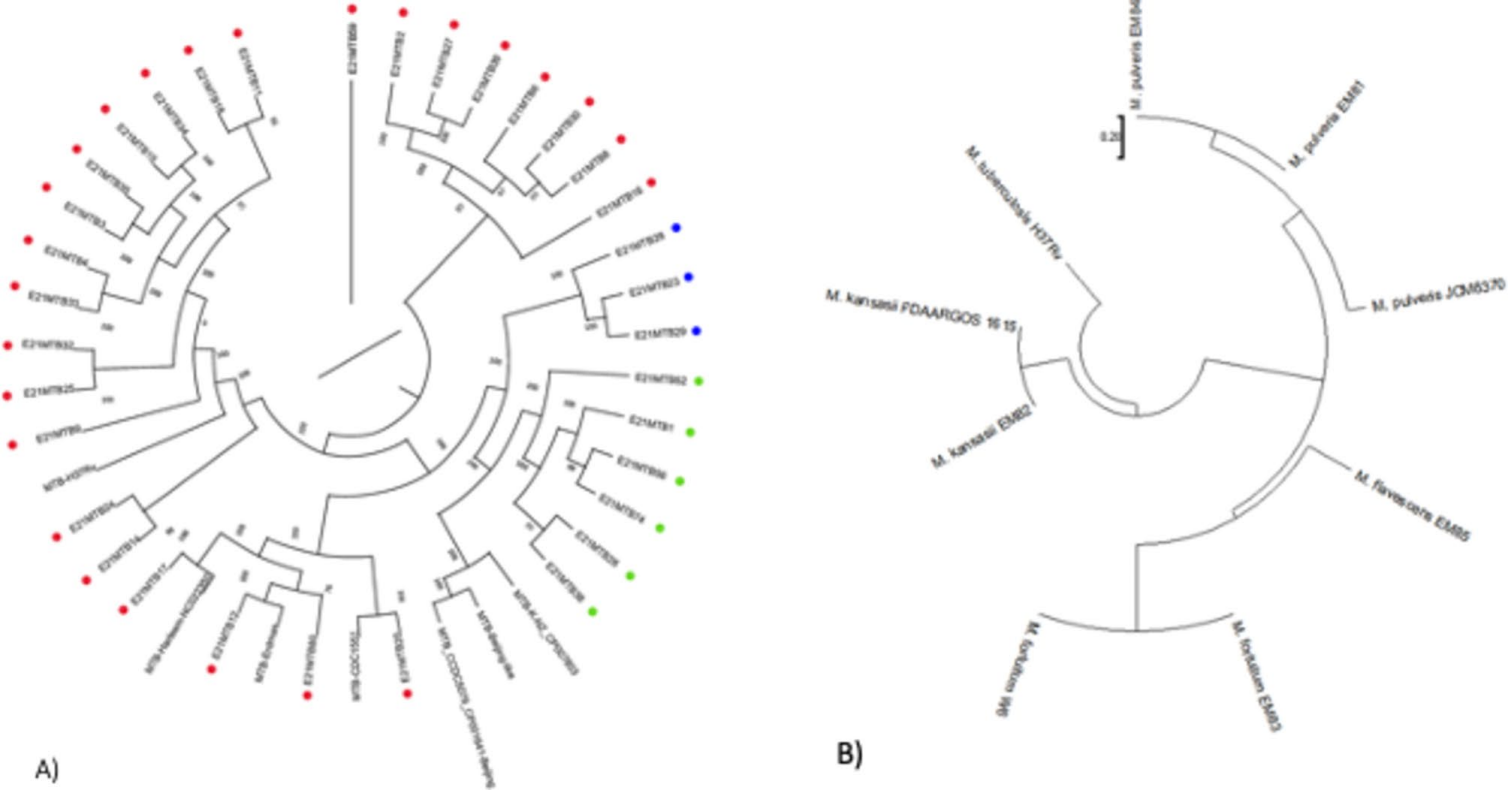
Whole genome sequence analysis of mycobacterial isolates from Nile Delta patients using Harvest suite with ParSNP alignment for building maximum likelihood tree. (**A**) A phylogenetic tree based on consensus sequence of each *M. tuberculosis* isolate assembled using *M. tuberculosis* H37Rv (NC_000962) as a templet. Some reference strains from different lineage were included in this tree. The number next to each branch are the shared split percentage with the reference (H37Rv). Red dots indicate lineage 4, green dots indicate lineage 3 while blue dots indicate *M. bovis*. (**B**) A phylogenetic tree of NTM isolates assembled using *M. tuberculosis* H37Rv (NC_000962) as an out branch. Some reference strains from the same species were included in this tree. The tree is scaled to represent the distance between different species and different isolates of the same species, scale is on the graph top left corner.

Sequenced *M. tuberculosis* isolates were assembled using the laboratory H37Rv strain (NC_000962.3) as a template. The consensus sequences for all isolates were used to build phylogenetic tree to show the overall relationship of isolates from the Nile Delta to worldwide isolates. As expected, all isolates belonging to lineages 3 and 4 clustered together and those belonging to *M. bovis* clustered together (Fig. 2A), another confirmation of the lineage assignment by earlier analysis. Despite PCR genotyping, isolates numbers 36, 52, 56, and 74 were clustered with lineage 3 with the presence of Beijing isolates representatives of lineage 2. Interestingly, isolates numbers 52 and 56 were assigned as lineage 3 by Mykrobe and TB profiler but contained *mtbk_20680*, a marker of Beijing strain as indicated by PCR genotyping.

For non-tuberculous mycobacteria (NTM) isolates, all sequenced whole genomes were aligned to their corresponding refence genomes (*M. fortuitum* CT6/W6, *M. pulveris* JCM6370, *M. kansasii* FDAARGOS1615) and created a phylogenetic tree representing the distances between those isolates and previously isolated NTM as well as the distance to *M. tuberculosis* H37Rv (Fig. 2B). The Egyptian isolates of NTM were closely related to other human NTM isolates isolated around the world. Interestingly, human isolate of *M. fortuitum* from Wisconsin (W6) and that from Egypt (EM83) were completely identical with whole genome alignment (data not shown).

### Drug resistance among isolates from the Nile Delta

The drug susceptibility test (DST) revealed that *M. tuberculosis* isolates that were mono-resistant to streptomycin, isoniazid, rifampin, and ethambutol (SIRE) represented 31%, 17.2%, 19.5% and 20.7% respectively (Table 1). A total of 47.1% of the isolates were sensitive to all four frontline anti-tuberculosis drugs while one isolate was resistant to all four anti-tuberculosis drugs combined. The lowest susceptibility rates were in Northern Delta governorates, except for mono-resistance to streptomycin which was higher (34.7%) compared to the Southern governorates (26.3%) (Supplementary Table 2). Overall, resistance phenotypes to the other anti-tuberculosis drugs (I, R, E) were similar between both North and South regions. Antibiotic susceptibility of NTM are shown in Table 2.

**Table 1:** Drug resistance phenotypes of *M. tuberculosis* isolates from the Nile Delta to frontline anti-tuberculosis drugs.

Phenotypic susceptibility	Number of isolates	Percentage (out of 87)
Pan-sensitive	41	47.1%
Streptomycin(S) resistance	27	31.0%
Isoniazid (I) resistance	15	17.2%
Rifampicin (R) resistance	17	19.5%
Ethambutol (E) resistance	18	20.7%
Monoresistance	33	37.9%
Double drug resistant (S and E)	9	10.3%
Multidrug resistant, MDR (I, R, E)	3	3.4%
MDR (S, I, R, E)	1	1.1%
Total number drug resistant isolates	46	52.9%

**Table 2:** Antibiogram for non-tuberculous mycobacteria.

Isolate ID	Clarithromycin	Amikacin	Streptomycin	Isoniazid	Tetracycline
*M. pulveris*	S	S	S	R	S
Seq pending	S	S	R	S	R
*M. Kansasii*	S	S	S	S	S
*M. fortuitum*	R	I	R	R	I
*M. pulveris*	R	R	R	R	R

*S* sensitive, *R* resistant, *I* inconclusive.

### Single nucleotide polymorphism associated with drug resistance

Whole genome sequencing was used to identify SNPs associated with resistance to any of the frontline anti-tuberculosis drugs (SIRE). Out of the 27 phenotypic resistant isolates to streptomycin, 12 showed related genotypic mutations: 11 in the *gid* gene and one in the *rpsl* (Supplementary Table 3). Two isolates showed double mutations in the *gid* gene simultaneously (p.Leu16Arg + p.Ser149Arg). For isoniazid, seven out of the 15 phenotypic resisters also showed genotypic mutations: four showed the well-established *katG* p.Ser315Thr mutation, one showed *mshA* p.Asn111Ser, another showed *fabG1* mutations and another showed the *katG* p.Arg463Leu. *katG* p.Arg463Leu, *ahpC* c. − 88G > A and *mshA* p.Asn111Ser mutations. Interestingly the two isolates with *fabG1* c. − 15C > T and *fabG1* c. − 47G > C were of the same lineage 4.6.2.2. For rifampicin, out of the 17 phenotypic rifampicin resistant isolates only six showed mutations in the *rpo* genes; two isolates in the *rpoB*, three isolates with mutation in the *rpoC* gene and one co-harboring mutations in both *rpoB* and *rpoC* (*rpoB* p.Asp435Phe + *rpoC* p.Arg69Pro). Out of the 19 phenotypic ethambutol resistance, 10 showed genotypic mutations (Supplementary Table 3) while six isolates showed mutations in *embB*, five isolates in *embC*, four in *embR* and one isolate in *embA*. Coincidence of multiple mutations was present in four isolates ranging from two to five mutations at once.

**Figure 3:**
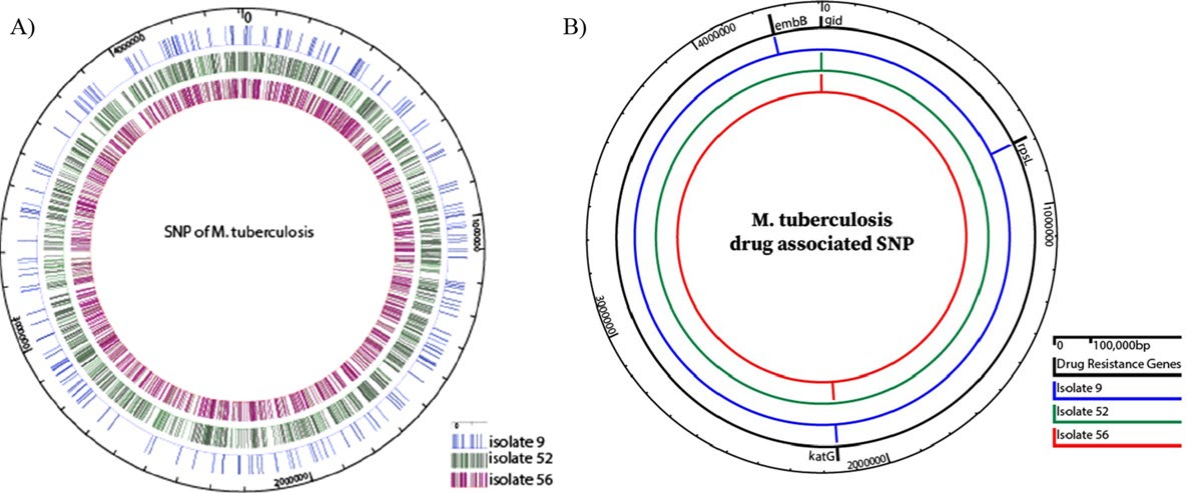
A circular map generated by GeneVision Software displaying whole single nucleotide polymorphisms (SNPs) in selected isolates with known drug resistance profile. (**A**) All SNPs in selected isolates. (**B**) SNPS related to drug resistance based on sequence analysis.

Taking advantage of the available whole genome sequences, we were able to identify SNPs for other anti-tuberculosis drugs not examined by the plate assay or MGIT system. For example, pyrazinamide genotypic resistance conferred the same *pncA* p.His57Asp mutation in five isolates in addition to one isolate with *pncA* c. − 33G > A mutation. The five isolates were *M. bovis* a natural resistant strain of pyrazinamide^24^. For ethionamide, mutations were identified in the *mshA* (n = 9), *FabG* (3), *ethR* (2) and *ethA* (1) genes. Finally, we compared the overall agreement between genotypic analysis of drug resistance and profiles identified in DST assays, the gold standard for antibiotic resistance (Supplementary Table 4). As shown, between 41 up to 100% of the established SNPs identified by sequence analysis were present in phenotypically resistant strains. Figure 3 shows all single nucleotide polymorphisms (SNPs) of three MDR isolates, among all the SNPs, only few are associated with drug resistance (Fig. 3). For second line drugs used to treat tuberculosis^25^; fluoroquinolone genotypic resistance was present in 78 isolates out of the total 87 isolates: all showed mutations in *gyrA*. Additionally, p.Glu21Gln was present in 77 isolates, p.Ser95Thr and p.Asn826Asp were present in the same 53 isolates. There were seven isolates with *gyrB* mutations and they were coinciding with *gyrA* mutations. Genotypic resistance to amikacin, capreomycin, kanamycin was also predicted based on three mutations in the *rrs* gene and two mutations in the *eis* genes^26^.

## Discussion

Tuberculosis and analysis of drug resistance strains of the causative agent *M. tuberculosis* remain an elusive goal for countries in the Middle East as a result of several technical and logistical difficulties. Earlier reports from Africa demonstrated the predominance of lineage 4^27^. Further, country-wise comparisons showed a correlation between genotypes associated with drug susceptible TB and drug resistant TB, implying that drug resistant TB is to a large extent, acquired by individuals within their respective African countries^28^. Similarly, the predominance of lineage 4 (83.9%) was observed in this study in the Nile Delta, followed by lineage 3 (11.4%). Among lineage 4 sub-lineage T was most predominant (26%) followed by sub-lineage LAM (15.6%) then Haarlem (11.9%). This profile was in agreement with a previous report from Egypt done by spoligotyping, where the majority of isolates (93.0%) belonged to lineage 4, including 44.3%, 13.4% and 10.8% of the ill-defined T clade, LAM and Haarlem families, respectively, and lineage 3 was identified in 7.0% of the isolates^7^. In addition, the majority of the isolates tested belonged to the T-1 family which is widely present in populations in all continents and corresponds to about 30% of all entries in the international database^29^. Egypt’s geographical location in the Mediterranean area could explain our findings. In Lebanon, among 60 isolates 13 patterns of *M. tuberculosis* complex family strains were identified: 41.6% of the strains belonged to the T 1 family, 25.0% to LAM 9, 10.0% to Haarlem 3, 3.3% to each of CAS, LAM 8, BCG and Family 36 and 1.7% to each of Haarlem 1, LAM 10, S, *M. africanum,* X 1 and T 3 families^30^.

The predominance of lineage 4 is in contrast to results of a previous study of Egyptian in 2018 that reported predominance of lineage 3 (76%)^11^. However, the isolates in the older study were collected from a localized geographic area, while our isolates were collected from widespread geographic locations. In general, *M. tuberculosis* in Egypt showed less diversity due to the relative homogeneity of communities and relatively stable population structure (sympatric pattern) where expat workers are rare^11^. Other countries in the middle east showed somewhat different patterns of lineage distribution. A study from Oman done on 70 genomes showed much more diverse *M. tuberculosis* population with four major lineages (L1, L2, L3, and L4) detected showing predominance of spoligotype EAI and Central Asian (CAS). The considerable variation and heterogony in Oman of lineages and spoligotypes is linked with the presence of a high percentage of non-Omani population coming from and frequently visiting high TB burden countries^31^. Interestingly, both non-tuberculous mycobacteria and *M. bovis* represented ~ 14% of the human isolates in this study, an indication of high prevalence of environmental and zoonotic mycobacteria in Egypt.

Drug-resistant tuberculosis (DR-TB) continues to be a public health threat both in Egypt and worldwide, among which, resistance to rifampicin (the most effective first-line drug) is of a greatest concern^3,6^. Resistance to rifampicin and isoniazid is defined as multidrug-resistant TB (MDR-TB) and both MDR-TB and rifampicin-resistant TB (RR-TB) require treatment with second-line drugs^1^. In this report, we examined the phenotypic and genotypic profiles of isolates of *M. tuberculosis* collected from the Nile Delta, Egypt and tested them against first line drugs. We also used genome-wide analysis to predict resistance to difficult to test anti-tuberculosis drugs (e.g. pyrazinamide) and second line drugs such as fluoroquinolones. In our analysis, the susceptibility to four drugs (S, I, R, E) was only 41.3%. In addition, among *M. tuberculosis* isolates, ~ 38% were resistant to only one drug, and only 1 isolate was resistant to all drugs tested. The low susceptibility rate is consistent with relapse and treatment failure reported earlier in Egypt^3,13^. Previously, our group reported that the rates of resistance to anti-tuberculous drugs were lower^11^, suggesting a temporal trend towards increased resistance rates over time, perhaps as a deleterious effect of the COVID-19 pandemic on the TB control programs during which the current study was conducted. Another difference between the two studies is about the source of the isolates; isolates from the current study were collected from wider geographic areas including Alexandria and Delta governorates that showed the highest rates of drug resistance^11^. The resistance level for mono rifampicin resistance (~ 19%) is higher than isoniazid (~ 17%), unlike several reports which shows inverse results. Because of the availability of most of antibiotics over the counter in Egypt, antibiotic resistance rates are higher than in other areas. However, this is not the case for anti-tuberculous drugs as they are provided by government hospitals for the registered patients only. This might explain the higher rates of rifampicin resistance as compared to the isoniazid as the former is widely available and provided in many forms to treat other infections while the latter is only given to registered TB patients^32^. Interestingly, other Middle East countries showed lower resistance rates to anti-tuberculous drugs including Saudi Arabia where resistant percentages were up to 5% only for streptomycin^11^. In a systematic review and meta-analysis on *M. tuberculosis* antibiotic resistance in Iran (2013–2020), the average resistance rates to SIRE were 10.6%, 6.9%, 7.9% and 5.7%^33^, those were much higher in Egypt (Table 1).

As expected, our genome-wide analysis identified predominant gene mutation markers associated with different anti-tuberculosis drugs including *rpoB* and *rpoC* for rifampicin; *katG* for isoniazid; *embBCR* for ethambutol; *pncA* for pyrazinamide; *gid* for streptomycin and *gyrA* for fluoroquinolones^25,26^. Although genome-wide analyses were useful to predict drug resistance to both first-line and second-line anti-tuberculosis drugs, the antibiotic sensitivity testing was not always in agreement with identified mutations. This disparity could arise from the limited set of reported mutations in the drug resistance genes, and other relevant mutations in these genes may be unreported^16,27^. In consistence with our results, phenotypic and genotypic patterns were poorly correlated in clinical multidrug-resistance *M. tuberculosis* isolates especially for ethionamide (agreement 56.4%)^34^. Although the WHO catalogue of mutations in *M. tuberculosis* has provided a valuable and comprehensive resource for genotypic data, yet there is still the need to identify an confirm additional drug resistance mutations, particularly for new and repurposed drugs. Moreover, Chen Y et al. reported DST phenotypic instability of *M. tuberculosis* strains adding justification to the phenotypic/genotypic discrepancies^35^.

A systematic review on African countries showed variations in strain predominance within the continent. lineage 4 predominates across Africa, while the Beijing genotype was found to be in some parts of Africa^28^, including two isolates identified correctly by WGS but not by multiplex PCR. This result highlights the sensitivity of the WGS over PCR genotyping for the determination of *M. tuberculosis* lineage assignment.

Regarding the drug-resistant TB, the Haarlem and CAS genotypes were predominantly associated with drug resistance in parts of North and East Africa while in Southern and West Africa the Beijing and LAM genotypes were highly associated with drug resistance. In contrast, the “ancient strains” such as *M. africanum* (MAF) strains are largely restricted to West Africa where these strains are mostly associated with drug susceptible TB. One possible reason for the stability of MAF in Ghana and West Africa might be adaptation of this lineage to specific human populations, as recent studies show a strong association of MAF with the Ewe ethnicity^28^. In conclusion, we report higher rates of resistance to *M. tuberculosis.* The COVID-19 pandemic could have contributed to the increased rates of drug resistance, compare to rate before the pandemic. Lineage 4 was the most prevalent (83.9%), followed by lineage 3 (11%). Among lineage 4, sub-lineage T was most predominant (26%) followed by sub-lineage LAM (15.6%) then Haarlem (11.9%). Although common mutations were detected in resistant isolates, the phenotypic resistance to anti-tuberculosis drugs in our isolates was not always associated with genotypic known mutations. It is undeniable that the NGS merit of generating big data is useful for the confident identification, lineage determination and relatedness establishment, yet the antibiotic resistance prediction is not well established due to the possible unreported mutations and/or sequencing errors. More genome wide genotypic and phenotypic comparisons are needed to identify the resistance markers and to understand the molecular mechanisms underlying the phenotypic instability to bridge this knowledge gap. Our study calls for reinforcement of the National Tuberculosis Program in Egypt to cope with the higher resistance rates, with a focus on governorates with higher rates of resistance. In addition, the study highlights the need to further strengthen the genotypic analyses of more drug resistant isolates of *M. tuberculosis* to improve their prediction accuracy.

### Supplementary Information

Supplementary Information.

## Data Availability

Sequences are available on the Genbank under Bioproject PRJNA1019495 Supplementary materials files.
