# Supplementary material for: Drug resistance and genomic variations among Mycobacterium tuberculosis isolates from The Nile Delta, Egypt
Source: Sci Rep. 2024 Sep 2;14:20401. doi: 10.1038/s41598-024-70199-8 (PMC11369133; doi:10.1038/s41598-024-70199-8)
Supplement: Supplementary file 1 — Supplementary Information. [file 41598_2024_70199_MOESM1_ESM.docx]

**Supplemental Table (1).** Lineage and spoligotype distribution among the isolates.(Total=87)

| Lineage | Sublineage | Family | Spoligotype | Number | Total (%) |
| --- | --- | --- | --- | --- | --- |
| Lineage 3 | Lineage 3 | East-African-Indian | CAS | 10 | 10 (11.4%) |
| Lineage 4 | 4.1.2.1 | Euro-American (Haarlem) | H1 Haarlem | 11 | 73 (83.9%) |
|  | 4.2.2.1 | Euro-American | LAM7 | 1 |  |
|  | 4.3 | Euro-American (LAM) | LAM | 11 |  |
|  | 4.6.2.2 | Euro-American | LAM | 3 |  |
|  | 4.4.1.1 | Euro-American | Orphans | 22 |  |
|  | 4.5 | Euro-American | T | 1 |  |
|  | 4.7 | Euro-American (mainly T) | T | 1 |  |
|  | 4.8 | Euro-American | T | 1 |  |
|  |  | Euro-American (mainly T) | T | 22 |  |
| M.bovis | M.bovis | M. bovis | Bov | 5 | 5 (5.7%) |

**Supplemental Table (2): Distribution of drug resistance phenotypes within the Nile Delta among 87 isolates (38 form Cairo laboratories and 49 form Alexandria).**

|  | **Number (%) of resistant isolates** | |
| --- | --- | --- |
| **Drug** | **Cairo** | **Alexandria** |
| **Streptomycin** | 10 (26.3%) | 17 (34.7%) |
| **Isoniazid** | 8 (21.1%) | 7 (14.3%) |
| **Rifampicin** | 10 (26.3%) | 7 (14.3%) |
| **Ethambutol** | 11 (28.9%) | 7 (14.3%) |
| **DDR (S & E)** | 4 (10.5%) | 5 (10.2%) |
| **MDR (I, R, E)** | 1 (2.6%) | 2 (4.1%) |
| **MDR (S, I, R, E)** | 0 (0.0%) | 1 (2.0%) |

**Supplemental Table (3):** List of gene mutations in established and potential drug resistance genes identified in *M. tuberculosis* whole genome sequencing from Greater Cairo and the Nile Delta.

| Antibiotic | Gene Related | Mutation/Aminoacid change | **WHO confidence Grading of mutation** | No of point mutations | |  |
| --- | --- | --- | --- | --- | --- | --- |
| Streptomycin | *gid* | c.102_102del | Gp2 | 1 | 11 | |
|  |  | p.Leu108Arg | Tier1 | 1 |  |  |
|  |  | p.Leu16Arg+ p.Ser149Arg | Gp5 | 2 |  |  |
|  |  | p.Leu16Arg | Gp 5 | 1 |  |  |
|  |  | p.Ala119Asp | Gp3 | 1 |  |  |
|  |  | p.His174Arg | Tier1 | 1 |  |  |
|  |  | p.Ala183Glu | Tier1 | 1 |  |  |
|  |  | p.His48Tyr | Gp1 | 1 |  |  |
|  |  | c.463_469del (frameshift) | Gp2 | 1 |  |  |
|  |  | p.Phe12Cys | Tier1 | 1 |  |  |
|  | *rpsl* | p.Lys88Arg | Gp 1 | 1 | 1 | |
| Isoniazid | *katG* | p.Ser315Thr | Gp1 | 4 | 6 | |
|  |  | p.Arg463Leu | Gp5 | 2 |  |  |
|  | *fabG1* | c.-47G>C | Tier1 | 1 | 2 | |
|  |  | c.-15C>T | Tier1 | 1 |  |  |
|  | *mshA* | p.Asn111Ser | Tier2 | 2 | 2 | |
|  | *ahpC* | c.-88G>A | Tier1 | 1 | 1 | |
| Rifampicin | *rpoB* | p.Asp435Phe | Gp1 | 1 | 3 | |
|  |  | p.Ser450Leu | Gp1 | 2 |  |  |
|  | *rpoC* | p.Gly594Glu | Gp5 | 2 | 4 | |
|  |  | p.Arg69Pro | Gp5 | 2 |  |  |
| Ethambutol | *embA* | p.Ser768Asn+ p.Ser788Cys+embA p.Leu792Val | Tier 1 | 1 | 1 | |
|  | *embB* | p.Pro92Ala | Tier 1 | 1 | 8 | |
|  |  | p.Val131Met | Tier 1 | 3 |  |  |
|  |  | p.Asn13Ser | Gp5 | 2 |  |  |
|  |  | p.Glu378Ala | Gp5 | 2 |  |  |
|  | *embC* | p.Val981Leu | Gp5 | 3 | 5 | |
|  |  | p.Thr270Ile | Gp5 | 2 |  |  |
|  | *embR* | p.Asp111Gly | Tier 2 | 1 | 4 | |
|  |  | c.-207C>G | Tier 2 | 2 |  |  |
|  |  | p.Ala176Gly | Tier 2 | 1 |  |  |
| Ethionimide | *fabG* | p.Ala225Val | Tier 1 | 1 | 3 | |
|  |  | c.-47G>C | Tier 1 | 2 |  |  |
|  | *mshA* | p.Asn111Ser | Gp 5 | 9 |  | |
|  | *ethA* | c.-22A>G | Tier 1 | 1 |  | |
|  | *ethR* | p.Met142Ile+ p.Gln143Lys | Tier 2 | 2 |  | |
| Kanamycin  Amikacin  Capreomycin | *rrs* |  | Tier 1 | 3 | 5 | |
|  | *eis* |  | Tier 1 | 2 |  |  |
| Fluoroquinolones | *gyrA* | p.Glu21Gln | Gp 5 | 77 | 195 | |
|  |  | p.Ser95Thr | Gp 5 | 53 |  |  |
|  |  | p.Gly247Ser | Gp 5 | 4 |  |  |
|  |  | p.Asp639Ala | Tier 1 | 1 |  |  |
|  |  | p.Gly668Asp | Gp 5 | 1 |  |  |
|  |  | p.Asn826Asp | Tier 1 | 53 |  |  |
|  |  | p.Arg607His | Tier 1 | 3 |  |  |
|  |  | p.Ala90Val | Gp 1 | 1 |  |  |
|  |  | p.Asp94Asn | Gp 1 | 2 |  |  |
|  |  |  |  |  |  |  |
|  | *gyrB* | p.Ala2Gly | Tier 1 | 1 | 6 | |
|  |  | p.Val137Ala | Tier 1 | 0 |  |  |
|  |  | p.Trp174 | Tier 1 | 1 |  |  |
|  |  | p.Ala403Ser | Gp 5 | 4 |  |  |
|  |  |  |  |  |  |  |

**Gp1**: associated with resistance, **Gp2**: associated with resistance-interim, Gp3: uncertain significance, **Gp5**: not associated with resistance. **Tier 1**: gene sequences that were considered most likely to contain resistance mutations. **Tier 2**: candidate genes, considered to have a lower, but still reasonable probability of containing resistance mutations.

**Supplemental Table (4):** Overall agreement between antibiotic resistance based on both phenotypic and genotypic analyses.

| **Antibiotics** | **No. Phenotype resistance** | **No. Genotype resistance** | **Mutation Association %** |
| --- | --- | --- | --- |
| Streptomycin | 27 | 12 | 44.4 |
| Isoniazid | 15 | 11 | 73.3 |
| Rifampicin | 17 | 7 | 41.2 |
| Ethambutol | 18 | 18 | 100.0 |

**Supplemental Table (5):** GenBank accession numbers of deposited whole genome sequence of isolates from this study.

| **Isolate Name** | **Total read count** | **Consensus length** | **% matched H37Rv** | **Ave. coverage** | **Accession number** |
| --- | --- | --- | --- | --- | --- |
| **E#1** | **2523854** | **4411493** | **88.6** | **100.71** | **JAVTLN000000000** |
| **E#2** | **2553897** | **4411537** | **89.72** | **102.45** | **JAVTLV000000000** |
| **E#3** | **2013324** | **4411527** | **87.87** | **79.34** | **JAVTMB000000000** |
| **E#4** | **1991017** | **4411478** | **82.59** | **78.78** | **JAVTMK000000000** |
| **E#5** | **1116843** | **4411514** | **69.98** | **42.2** | **JAVTMM000000000** |
| **E#6** | **2662033** | **4411570** | **89.64** | **105.07** | **JAVTMQ000000000** |
| **E#8** | **2759804** | **4411513** | **92.09** | **106.38** | **JAVTNC000000000** |
| **E#9** | **1299947** | **4411510** | **69.39** | **55.44** | **JAVTNE000000000** |
| **E#11** | **2550594** | **4411516** | **82.88** | **103.38** | **JAVTLO000000000** |
| **E#12** | **2327276** | **4411520** | **80.61** | **91.76** | **JAXIQC000000000** |
| **E#13** | **720363** | **4411537** | **56.18** | **30.96** | **JAVTLP000000000** |
| **E#14** | **4208949** | **4411462** | **91.4** | **144.42** | **JAVTLQ000000000** |
| **E#15** | **2280952** | **4411535** | **90.47** | **83.58** | **JAVTLR000000000** |
| **E#16** | **2684067** | **4411534** | **91.09** | **96.15** | **JAVTLS000000000** |
| **E#17** | **2279675** | **4411523** | **82.71** | **82.7** | **JAVTLT000000000** |
| **E#18** | **2406606** | **4411519** | **74.45** | **81.15** | **JAVTLU000000000** |
| **E#20** | **2514703** | **4411482** | **87.69** | **91.71** | **JAVTLW000000000** |
| **E#21** | **599059** | **4411519** | **51.83** | **20.23** | **JAVTLN000000000** |
| **E#23** | **3007006** | **4411466** | **85.36** | **110.34** | **JAVTLX000000000** |
| **E#24** | **2373758** | **4411463** | **85.83** | **87.69** | **JAVTLX000000000** |
| **E#25** | **1694338** | **4411471** | **84.46** | **60.5** | **JAVTLY000000000** |
| **E#26** | **1743836** | **4411485** | **76.21** | **64.85** | **JAVTLZ000000000** |
| **E#27** | **3404481** | **4411492** | **77.86** | **105.83** | **JAVTLZ000000000** |
| **E#28** | **4078704** | **4411516** | **68.51** | **121.07** | **JAXIQB000000000** |
| **E#29** | **2224968** | **4411493** | **73.59** | **80.48** | **JAVTMA000000000** |
| **E#30** | **3618244** | **4411546** | **67.32** | **129.68** | **JAVTMC000000000** |
| **E#31** | **1265266** | **4411476** | **63.03** | **38.74** | **JAVTMD000000000** |
| **E#32** | **2022767** | **4411507** | **73.99** | **71.8** | **JAVTME000000000** |
| **E#33** | **1755487** | **4411520** | **69.44** | **65.95** | **JAVTMF000000000** |
| **E#34** | **1878219** | **4411490** | **71.17** | **67.61** | **JAVTMG000000000** |
| **E#35** | **2056943** | **4411538** | **85.91** | **73.61** | **JAVTMH000000000** |
| **E#36** | **1569736** | **4411546** | **80.12** | **57.34** | **JAVTMI000000000** |
| **E#37** | **1183078** | **4411516** | **78.41** | **44.21** | **JAXIQG000000000** |
| **E#38** | **692127** | **4411561** | **28.48** | **25.47** | **JAVTMP000000000** |
| **E#39** | **2794203** | **4411512** | **78.44** | **95.77** | **JAVTMJ000000000** |
| **E#40** | **1253020** | **4411555** | **77.1** | **48.02** | **JAVTML000000000** |
| **E#52** | **1965458** | **4411615** | **98.55** | **104.88** | **JAXIQH000000000** |
| **E#56** | **1840414** | **4411587** | **98.00** | **96.34** | **JAXIQI000000000** |
| **E#74** | **2152278** | **4411525** | **98.63** | **115.88** | **JAVTMY000000000** |
| **E#80** | **2104330** | **4411643** | **98.39** | **110.92** | **JAVTND000000000** |
| **EM#81** | **1895892** | **5313305** | **90%** | **83** | **SAMN38977419** |
| **EM#82** | **1962234** | **6572050** | **100%** | **70** | **SAMN38977420** |
| **EM#83** | **972048** | **6688155** | **100%** | **33** | **SAMN38977421** |
| **EM#84** | **560326** | **5311123** | **94%** | **24** | **SAMN38977422** |
| **EM#85** | **3616368** | **5075854** | **96%** | **160** | **SAMN38977423** |


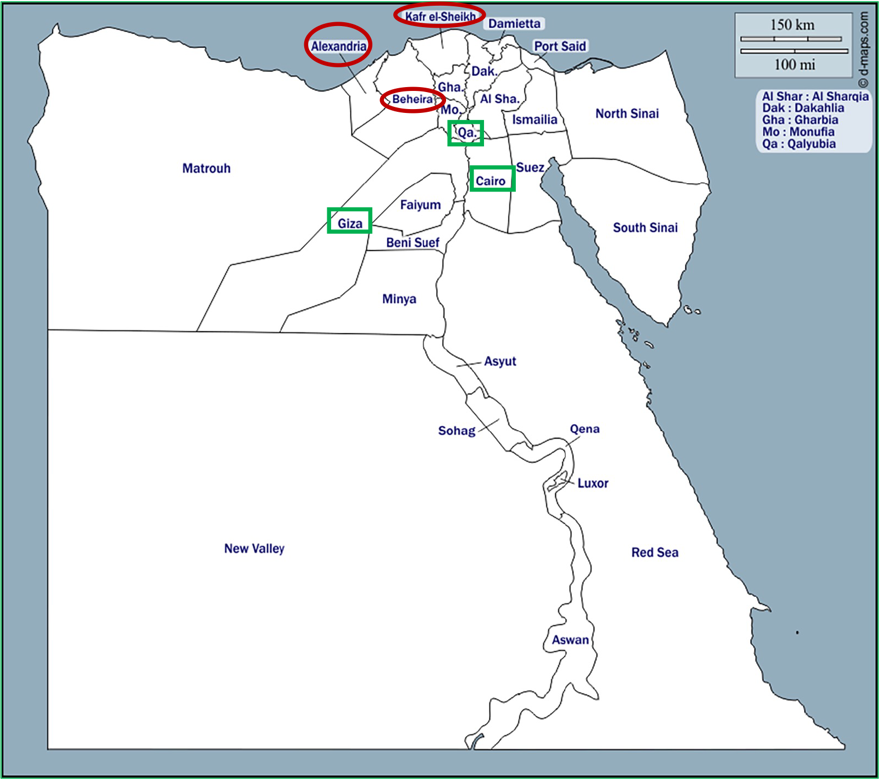


**Supplemental Fig. 1:** A map of Egypt with the Nile Delta showing the Northern (red circles) and Southern (green boxes) governorates. A total of 102 isolates were received from the Nile Delta (51 isolates each from Alexandria and Cairo central laboratories). Map source: <https://en.m.wikipedia.org/wiki/Subdivisions_of_Egypt#Governorates>


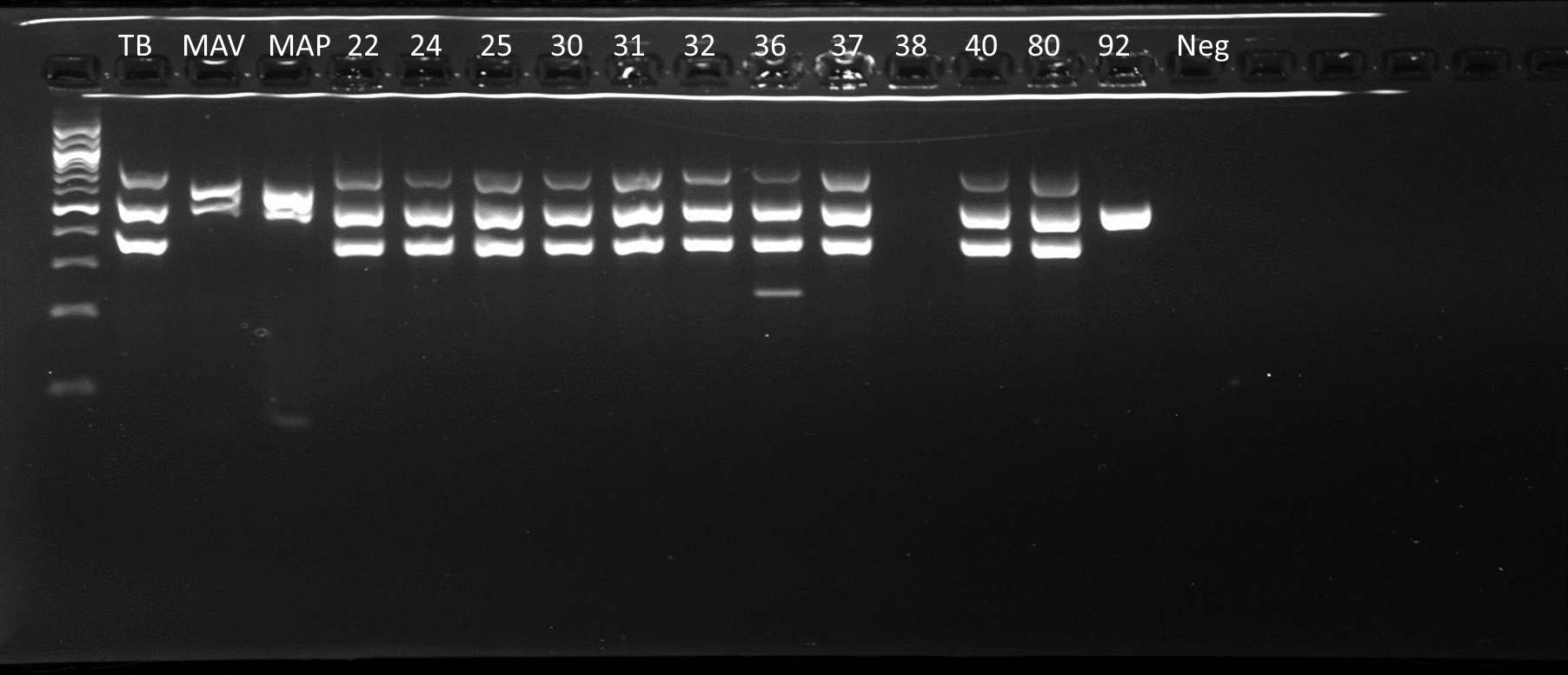


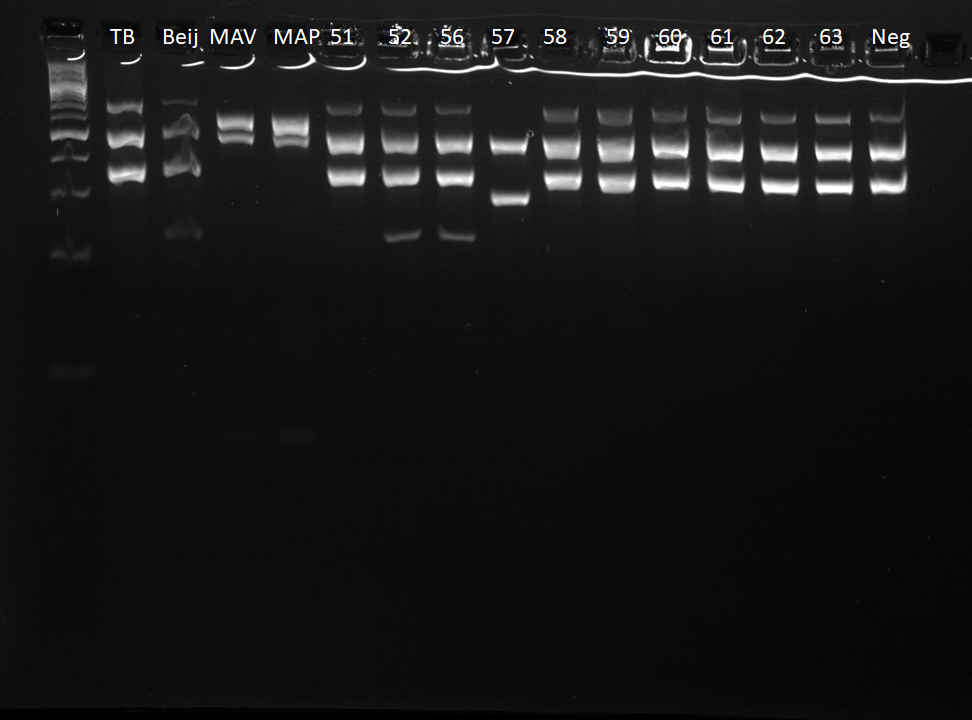


**Supplemental Fig. 2.** Beijing clade among *M. tuberculosis* from the Nile Delta isolates. PCR genotyping of *M. tuberculosis* isolates from Egypt. Note the extra bands present for isolates numbers 36, 52 and 56 indicating their genotype as members of the Beijing clade of M. tuberculosis.
